# Racial and Ethnic Differences in Perceptions of, and Barriers to, Cost-of-Care Conversations Among Older Adults in Florida

**DOI:** 10.1093/geroni/igaa057.265

**Published:** 2020-12-16

**Authors:** Kyaien Conner, Jacqueline Wiltshire, Erica Anderson, Edlin Garcia Colato, Iraida Carrion, Barbara Orban

**Affiliations:** 1 University of South Florida, Tampa, Florida, United States; 2 University of South Florida, Garden Grove, California, United States; 3 University of South Florida, Tampa, United States

## Abstract

Cost of care conversations (CoC) between patients and doctors have been shown to lower overall healthcare and patients’ costs. How, it is unclear why CoC are not occurring more frequently among high cost patients such as older adults. To address this important question, we conducted three race-stratified focus groups (n=10 Whites, n=9 African Americans, and n=8 Latino/Hispanics) to assess perceptions about, and barriers to, CoC in a convenient sample of adults ages ≥65 from Adult Centers in Tampa Bay, Florida. An inductive content analysis approach was utilized by research team members to analyze qualitative data. Findings indicated that CoC are not occurring. White participants perceived that CoC were not occurring because they did not have issues paying for care. African Americans perceived that CoC were not occurring because doctors are not trained to understand finances, insurance, and medical billing. Latinos/Hispanics perceived that doctors are meant to take care of patients, and receptionists, administrators and billing departments should handle CoC. Wait time and perceived stress/rush of doctors were identified as CoC barriers for whites, while doctors’ attitude was a barrier for Blacks/African Americans, and perceptions about CoC being “taboo” was a major barrier for Latinos/Hispanics. Overall, participants indicate that it is easier to have CoC if they had developed a good rapport with the doctor, had confidence in the doctor, and felt the doctor was interested in and cared about them. The findings suggest that promoting CoC among older adults will require addressing social and cultural concerns of racial/ethnic minority groups.

